# Social status qualifiers: dimensions and determinants of factors shaping social status for women and men in Sweden

**DOI:** 10.3389/fsoc.2023.1264896

**Published:** 2024-01-11

**Authors:** Ylva Ulfsdotter Eriksson, Bengt Larsson

**Affiliations:** ^1^Department of Social Studies, Linnaeus University, Växjö, Sweden; ^2^Department of Sociology and Work Science, University of Gothenburg, Gothenburg, Sweden

**Keywords:** social status, status qualifiers, status dimensions, postmaterialism, gender

## Abstract

This study contributes to our understanding of what lifestyle factors affect the social status of women and men in contemporary postmaterialist societies. We examine the dimensions and determinants of social status qualifiers among Swedish people using a survey of 1,650 Swedish respondents who ranked the importance of 14 qualifiers for the social status of a woman and a man. The analysis showed surprisingly strong similarities in what factors affect the social status of women and men – both in the importance of individual status qualifiers and in the three underlying status dimensions: The highest-ranked dimension included status qualifiers related to external material resources and properties. The second most important dimension comprised interactional resources such as manners, looks, being married and having children. The third dimension concerned the importance of interest and engagement in politics, the environment, and fine art, which were of the least importance for social status. The few significant differences in ascriptions of status for a woman or a man were rather gender stereotypical. In addition, the analysis revealed some significant differences in status perceptions among the respondents: Gender, class, educational background, and country of birth were among the main determinants of such differences.

## 1 Introduction

Throughout societies, people create and reproduce social status orders through the valuation of individuals, objects, and practices. Social status is a “fundamental feature of social life” (Leary et al., 2014, 159), and when playing out in interpersonal social relations, social status is ascribed, or recognized, by others. Consequently, people put effort into highlighting features that others can recognize. But, how are such status cues valued by others? And are the same status cues equally important for women and men? In this article, we explore common status qualifiers to discern their importance in accruing social status, and whether these features are of different importance for a woman and a man.

Status cues, or qualifiers, can be instrumental social values, such as possessions and personal characteristics, or relational, like social networks and relationships (Berger et al., 1998; Leary et al., 2014). Hence, social status is present in all aspects of our social life (Ridgeway, 2019): the occupation and the education we have, the car we drive, the type of housing and the neighborhood we live in, the clothes we wear, the food we eat and the restaurants we visit, the places we travel to and the experiences we make, our looks and behaviors, and in many other aspects of life (Goffman, 1969; Berger et al., 1998; Anderson et al., 2001; Carr and Vignoles, 2011; Anderson and Cowan, 2014; Blaker and van Vugt, 2014; Ridgeway, 2019; *cf.* Söderqvist-Tralau, 2009). Even though entities may be ranked in terms of status, such as social groups (Weber, 1970; *cf.* Turner, 1988), and organizations (Podolny, 2005; Sauder et al., 2012), *social status* usually connotes prestige hierarchies among individuals and their lifestyles (Wegener, 1992; Bauman, 2007; Reckwitz, 2021). Thus, everyday elements are assessed and valued as aspects of status. They are also used to create or claim social status in interpersonal social settings. Social status is “ubiquitous in social life” (Anderson et al., 2001, 116).

As a “social ranking of people, groups, or objects in terms of social esteem, honor, and respect” (Ridgeway, 2019, 1), social status correlates not only with lifestyles and sense of worthiness but also with subjective well-being, health, and stress (Wilkinson and Pickett, 2009; Fujishiro et al., 2010; Anderson et al., 2012), status anxiety and striving (De Botton, 2008; Anderson et al., 2015; Kim and Pettit, 2015), choice of spouse (Whitbeck and Hoyt, 1994), and political attitudes (Chan et al., 2011). Social status relates to and has consequences similar to social class. This connection was already obvious in Weber’s distinction between class and status groups, in which the former stratification was based on “relations of production and acquisition of goods” whereas the latter concerned certain lifestyles relating to an individual’s social esteem, honor, and privileges based on education, profession, or descent (Weber, 1970, 183ff; Weber, 1978, 302ff.). Social status may thus connote both older stratification principles based on honor and newer ones related to the individualization of lifestyles in late modern consumer societies (Turner, 1988; Bauman, 2007).

Bourdieu’s *Distinction* (1984) was an attempt to “rethink Max Weber’s opposition between class and *stand*” through the concepts of economic and cultural capital in an empirical analysis of lifestyles in France in the 1960s (Bourdieu, 1984, xii). Even though the general reproduction mechanisms in his work have been confirmed in other countries, the question of social status dimensions and determinants in contemporary societies has not been exhausted (Bourdieu and Wacquant, 1992, 78 f.; *cf.* Atkinson, 2018). Just as the structure of social classes exhibits both stability and change as society transforms, so does the social status structure. Social status may even be more important than class in late modern societies because lifestyles and consumer identities are becoming more individualized and fluid, and more constitutive of individual identities and life prospects (Beck and Beck-Gernsheim, 2002; Bauman, 2007; Reckwitz, 2021).

This study contributes to our understanding of lifestyle factors’ effect on individuals’ social status in contemporary welfare societies by exploring how a selection of common status qualifiers is perceived as significant for the social status of women and men in Sweden. Gender differences are of particular interest because there is a tendency to overlook that aspect in social status studies (Atkinson, 2018), and the Swedish case is interesting because of its extreme position in terms of postmaterialist values and gender equality (Inglehart, 2018; WEF, 2019). Drawing on quantitative survey data, *the study aims to explore the dimensions and determinants of social status perceptions* by addressing the following questions: What status qualifiers are important in the social status of a woman and a man in contemporary Sweden? What are the underlying dimensions of these status qualifiers? Are there any group differences (e.g., gender, age, education, or class) in the social status qualifiers and dimensions that are emphasized as important for the social status of women and men? This study also indirectly contributes to the discussion of Sweden as an example of a postmaterialist or even postmodernist consumer society (Söderqvist-Tralau, 2009; Inglehart, 2018; Ulver, 2019).

The article continues with a discussion of how we conceive social status in relation to the literature, followed by a discussion of relevant status qualifiers in the Swedish context. Next, a description of the materials and methods, which is followed by the findings. We then conclude with a discussion.

## 2 Social status in theory and research

### 2.1 Conceptualizations of social status in the literature

Social status is a vague and multifaceted notion, conceptualized differently depending on the discipline and research focus (Turner, 1988; Wegener, 1992; Leary et al., 2014). In sociology, the conventional starting point for a discussion of social status is Weber, who in *Class, Status, Party* (Weber, 1970, 194) introduced the concept of status to distinguish between the economic and social order in society: “one might thus say that ‘classes’ are stratified according to their relations to the production and acquisition of goods; whereas ‘status groups’ are stratified according to the principles of their consumption of goods as represented by special ‘styles of life’“. However, the boundary between the economic and social spheres is not conclusive, as lifestyles depend on and relate to economic conditions. In *Economy and Society* (1978), it is also emphasized that lifestyles are based on education, profession, and ancestry. This close connection between economy and ways of life has been thoroughly explored by Bourdieu, which is discussed below. However, first, we report on some theoretical distinctions and previous research to specify our perspective on and operationalization of social status.

In empirical research, “objective status” and “socioeconomic status” (SES) are descriptive terms for an ordinal scale of stratification of individuals based on occupation, income, and education (Oesch and Vinga, 2022). SES is often contrasted with “subjective social status” (SSS), defined as the “social respect or esteem people believe is accorded them within the social order” (Gidron and Hall, 2017, 61), or as how individuals perceive their social positions relative to others on the MacArthur scale (*cf.* Chiang et al., 2021). Whereas SSS implies self-assessments related to such a collective status order, SES generally measures the economic resources of individuals (including human capital). Neither concept captures social status in terms of lifestyles, based on the collective valuation of individuals, objects, and practices, ranking them at various levels of esteem, honor, privilege, reputation, respect, or prestige.

Some streams of research focus more limitedly on social rankings of occupations (Treiman, 1977; Ganzeboom and Treiman, 1996; Chan and Goldthorpe, 2007; Ulfsdotter Eriksson and Nordlander, 2024), organizations (Podolny, 2005; Sauder et al., 2012), or cultural consumption practices (Chan, 2011; Atkinson, 2018). Within the social-psychological strand of research, Leary et al. (2014) discussed the displaying of symbols to pursue social status. High status is, for instance, signaled by high-end possessions, such as expensive brands in clothes and cars, and ownership of a nice house (*cf.* Carr and Vignoles, 2011). People may also communicate social status through certain manners and behaviors. In contrast to our study, the focus in some of the more psychologically oriented research is on how people strive to signal or gain status and not how such status cures are evaluated in general (*cf.* Berger et al., 1998).

Other strands focus on the ranking, closure, and cohesion in standing or esteem of status groups based on gender, age, or skin color (Ridgeway, 2019). While these studies contribute to our understanding of social status, they lack aspects of the multifaceted approach in Weber’s conceptualization that relate to lifestyles and prestige. As stated by Turner (1988, 5):

A status is a position within the social structure by which an individual … is evaluated by reference to prestige or honor…. This evaluation will be both personal and objective, in that one’s self-evaluation is closely related to the external evaluation that one receives from significant others according to one’s location in a social hierarchy. Within the sociological literature, we have identified a “subjective” dimension of status (individual perceptions of prestige) and an “objective” (the socio-legal entitlements of the individual).

This distinction, between subjective and objective status, differs from SES or SSS. Whereas SES pulls the concept of objective status toward class stratification, Turner (1988) pulls it toward entitlements, rights, and polity. In our conceptualization, however, social status leans more toward what Turner calls the cultural and “subjective” aspect of status. However, we find it misleading to define this as “subjective,” as it consists of collective valuations of individuals, objects, and practices, objectively giving them various levels of esteem, honor, privileges, reputation, respect, or prestige (Leary et al., 2014; Anderson et al., 2015; Ridgeway, 2019).

In addition, whereas Turner (1988, 5) prefers to view status as an attribute of groups, we focus on individuals. The reason is that in late modern consumer societies, status has become less firmly anchored in cohesive groups or categories, according to theories claiming that lifestyles and status orders are more plural than previously (Bauman, 2007; Milner, 2019). This does not imply “the end of status” (Turner, 1988, 76), or as postmodernists claim, that “everyone can be anyone” (Featherstone, 1991, 83; *cf.* Campbell, 2004). On the contrary, late modern societies seem to increase individual responsibility for creating and upholding status in the competition for “singularity” – that is, social distinction (Reckwitz, 2021).

### 2.2 Status qualifiers – the Swedish context and previous research

Sweden is no longer the epitome of the egalitarian social democratic welfare state it once was. Clear neoliberalization tendencies and increasing economic inequalities have emerged since the 1980s (Larsson et al., 2012; Therborn, 2020). As shown by both Chancel et al. (2022) and Berglund (2024), rising economic inequalities are due to capital income, social transfers, and tax rates, rather than wages (i.e., paid labor). There is also a trend for objective class identity to have a diminishing effect on subjective identity because “a majority of the Swedish population locate themselves in the middle of the social structure” (Karlsson, 2017, 1057). In terms of cultural values, Sweden has long since scored extremely high on postmaterialism, both in terms of self-expression and secular–rational values (Inglehart, 2018).

Against the background of a tradition of “statist individualism” (Berggren and Trägårdh, 2022), and with the added effect of the welfare state as “an experimental apparatus for conditioning ego-related lifestyles” (Beck and Beck-Gernsheim, 2002, 28), the increased of inequalities and postmaterialist values have turned Sweden into a society of individuals. Some claim that it is a hyper-individualistic society, in which consumer trends have an immediate impact “in a very broad middle class apt for everyday status competition” (Ulver, 2019, 56). However, the comparative European literature does not confirm that status-seeking behavior is exceptionally high among the Swedish population (Delhey et al., 2022).

In this context, it is not surprising that status issues are commonly raised in Swedish media (e.g., Söderqvist-Tralau, 2009). Suggestions about sources of social status are numerous: money, consumption, style and looks, education, a prestigious occupation, or just success or self-realization. There is often a postmodernist influence in claims that social status is based on self-creation and consumerism (Ulver, 2012, 44 f.; *cf.* Bauman, 2007). Then again, studies show marked stability and correspondence with international studies on how occupations are ranked by prestige in Sweden (Svensson and Ulfsdotter Eriksson, 2009). All these findings raise questions regarding the importance of “modern” status qualifiers, such as occupation, compared with late modern consumerist status cues, not least because postmaterialist values imply that traditional status symbols are not very important (*cf.* Delhey et al., 2022).

As shown in both sociological and psychological studies, possible social status cues based on lifestyles are practically innumerable (Berger et al., 1998; Fişek et al., 2005; Leary et al., 2014; Anderson et al., 2015). In this study, we elaborate on a broad set of status qualifiers mirroring different kinds of resources that may, or may not, be associated with social status. Following the dimensions distinguished in Bourdieu’s seminal analysis (1984; *cf.* 1986), we initially discuss them in relation to economic, cultural, and social resources.

Economic status qualifiers, such as money and financial assets, are surely related both to class and status. Such assets provide purchasing power to realize a high-status lifestyle. However, money has a cultural value that stands “over and above its expenditure for articles of consumption or its use for the enhancement of power,” that is, money confers social status in itself (Merton, 1968, 190; *cf.* Carr and Vignoles, 2011). When invested in objects and practices, financial resources are also transformed into cultural status qualifiers (i.e., objective cultural capital), such as durable goods like houses, cars, or boats, which indicate both economic resources and lifestyle (Bourdieu, 2005). Where and how one lives also signals social status because different residential areas have different reputations in addition to various types of housing (Birenbaum, 1984; Bridge, 2001; Leary et al., 2014).

Educational degrees and occupation provide status *per se* (i.e., institutionalized cultural capital), while also being associated with economic resources. A person’s occupation is considered an important aspect of status because it positions individuals in the economic order (Treiman, 1977; Ganzeboom and Treiman, 1996). There are also cultural qualifiers related to personal appearance (i.e., embodied cultural capital): looks, clothing style, manners, and speech, which have been discussed as status cues (Fişek et al., 2005). Extroverted, sociable, and physically attractive people are ascribed high status (Anderson et al., 2001; Haas and Gregory, 2005; Frevert and Walker, 2014), and speech may be related to both status and stigma, such as having a dialect, a foreign accent, or limited vocabulary compared with being well spoken (Bourdieu, 1991; *cf.* Goffman, 1969; Birenbaum, 1984).

As shown by Bourdieu (1984), status may be assigned to interest and connoisseurship in relation to sophisticated culture and fine art, such as literature, classical music, and antiques (*cf.* Atkinson, 2018). There may also be forms of cultural capital and even stigmatizing effects related to ancestry and kin, as indicated by surnames, which in Sweden vary from common patronyms to those of the bourgeoisie or traditional nobility to foreign-sounding names (Goffman, 1969; *cf.* Clark, 2012). Another family-related status qualifier is that of being married and having children.

Having many friends and a vast social network may also be a sign of social status (i.e., social capital). That is, interactional and relational aspects are important status cues (Leary et al., 2014). People in high social status positions are often considered attractive (Blau, 1960) and “social networks are also important … [for] physical safety, good health, companionship, social esteem, etc.” (de Graaf and Flap, 1988, 453). Today, individuals also signal and accumulate status through digital social networking and self-presentation on social platforms (Reckwitz, 2021).

There have been suggestions of a specific form of cultural capital in Sweden that emerged in the early popular movements and was reinforced in the egalitarian culture of student councils, youth organizations, trade unions, and board memberships. Such “organizational capital” is based both on the display of certain civic values and the ability to navigate in organizations and function as a spokesperson (Broady, 1985, 1998, 13f; Häuberer, 2014). Given the postmaterialist value tendencies in Sweden, one might also suspect status gains from engagement in nature and the environment.

Matters of how an individual acts to signal status were discussed with a more pronounced interactionist perspective by Berger et al. (1998; *cf.* Fişek et al., 2005). Focusing on status cues, they distinguished, on the one hand, between tasks (what someone does, i.e., acts and behaviors) and categories (what one is or is ascribed to be, i.e., identity matters), and on the other, whether the status cues was indicative or expressive. Berger et al. (1998, 159) thus suggest that “different types of cues operate in combinations with each other.” In our interpretations of the findings, we have been inspired also by this approach.

With this overview of social status concepts and qualifiers/cues, we have laid the ground for the operationalization of social status, while arguing that individual social status is an important research area. That is, even if concepts such as “objective” and “subjective” status, and “social class” and “group status” are relevant for contemporary status stratifications, there are also important cultural aspects of social status not exhausted by such approaches.

## 3 Materials and methods

This study is based on a survey distributed by Statistics Sweden in 2018 to 7,000 randomly selected respondents. Drawing on 1650 unique answers (23.6% response rate), this article explores two questions focusing on social status: “What importance do you consider the following factors have for an adult [Q1: man’s] / [Q2: woman’s] social status in Sweden?” Both questions included a list of 14 items based on the abovementioned status qualifiers (Table 1). The response alternatives were assessed on a 5-point Likert-type scale (0 = “of no importance,” 1 = “of rather little importance,” 2 = “of some importance,” 3 = “of fairly great importance,” 4 = “of great importance”), see Table 2.

**Table 1:** Status qualifiers.

**Items**	**Short name**
How much money and other financial assets they have	Money/assets
What kind of residential area they live in	Residential area
What type of housing they have	Housing type
What other property they have	Other property
What occupation they have	Occupation
What kind of education they have	Education
Their looks and clothing style	Looks/style
Their ancestry/kin and/or family name	Ancestry/family name
Whether they are married and have children	Married/children
How many friends/how large a social network they have	Friends/social network
The way they behave and talk	Manners/speech
Their participation in voluntary associations and politics	Associations/politics
Their interest in nature and the environment	Nature/environment
Their interest in e.g., art, literature, music, antiquities	Fine art/antiquities

The explorative analyses were conducted in five steps. First, principal component analyses (PCA, Varimax rotation) were performed separately for the 14 items on women’s/men’s social status qualifiers. The solutions, based on the Kaiser criterion (eigenvalue >1), produced three factors for women’s status qualifiers (Table 3) but only two for men’s status qualifiers. Based on the theoretical interpretation of the dimensions and the scalability of the items, we also chose a solution with three dimensions for men’s status qualifiers (Table 4; *Cf.* Kim and Mueller, 1978).

**Table 4:** Men’s status dimensions.

	**Mean**	**F1**	**F2**	**F3**
	0–4			
Manners/speech	2.80		**0.514**	
Occupation	2.77	**0.781**		
Money/assets	2.76	**0.828**		
Residential area	2.60	**0.838**		
Housing type	2.53	**0.849**		
Education	2.46	**0.587**		
Other property	2.45	**0.817**		
Looks/style	2.34		**0.636**	
Friends/social network	2.11		**0.752**	
Associations/politics	1.67			**0.671**
Nature/environment	1.57			**0.815**
Ancestry/family name	1.52	0.480	**0.439**	
Married/children	1.48		**0.718**	
Fine art/antiquities	1.45			**0.804**
**Grand mean**	2.18	2.60	2.05	1.56
Eigenvalue		5.60	2.20	0.96
% of variance explained		40.0	15.7	6.9
Cronbach’s α (bold)		0.90	0.72	0.73

Ancestry/family name was included in the scale for Index 2 on men’s social status, even though it loaded more strongly on Factor 1.

Means and factor loadings (*n* = 1,572).

**Table 3:** Women’s status dimensions.

	**Mean**	**F1**	**F2**	**F3**
	0–4			
Manners/speech	2.98		**0.611**	
Looks/style	2.80		**0.753**	
Occupation	2.79	**0.751**		
Education	2.70	**0.646**		
Residential area	2.51	**0.820**		
Money/assets	2.49	**0.818**		
Housing type	2.46	**0.814**		
Friends/social network	2.38		**0.774**	
Other property	2.09	**0.781**		
Married/children	1.92		**0.779**	
Associations/politics	1.81			**0.727**
Nature/environment	1.81			**0.869**
Fine art/antiquities	1.72			**0.807**
Ancestry/family name	1.66		**0.552**	
**Grand mean**	2.29	2.51	2.35	1.78
Eigenvalue		6.37	1.90	1.09
% of variance explained		45.5	13.5	7.8
Cronbach’s α (bold)		0.91	0.83	0.80

Means and factor loadings (*n* = 1,568).

Second, means (range 0–4) were calculated for all items, and grand means for all the items loading strongly (in bold) on the factors. The means were used to rank all 14 items in rows from high to low. The grand means were used to show the order of the factors in the columns (Tables 3 and 4).

Third, a rank correlation (Spearman’s ρ) was performed on the rank orders of qualifiers for the social status of women and men. Thereafter, the difference between the means of each status item for women and men was compared to identify those with marked gender differences. As the means on 10 of the 14 items were higher for a woman’s status than for a man’s, the means were also divided by the grand means for each gender to standardize the scales and clarify the main differences (data not shown).

Fourth, six standardized additive indexes (range 0–4) were created from the PCA (Tables 3, 4). We used identical indexes for the social status of women and men, even though the item “Ancestry and family name” was loaded in a slightly different way for men (Table 4). This item was included in Index 2 to improve the comparability of the regressions after we checked that this only marginally changed the scalability of indexes for men and after checking that alternative indexes did not affect the regression results significantly.

Finally, the indexes were used as dependent variables in ordinary least squares (OLS) regressions to explore respondents’ perceptions of the status dimensions (Tables 3, 5). The independent variables were age, sex, education, country of origin, resident municipality, and subjective class background, that is, the class context in which the respondent was raised. The reason for choosing this class variable instead of the objective class was that we, in line with Bourdieu’s analyses (1984; 1986), believe that an individual’s basic values regarding status and lifestyle are formed during childhood and early youth. In addition, the education variable is a relatively good proxy for the individual’s current class position. We included the variable “my occupation’s prestige” as previous studies have shown that the prestige of one’s occupation has an impact on perceptions (Alexander, 1972; Ulfsdotter Eriksson and Nordlander, 2023). We report the standardized β coefficients because our focus was on comparing effects across the six indexes (Table 5). We also ran regressions on all the individual items to understand better the effects on the indexes (data not shown).

**Table 2:** Dependent and independent variables in OLS regressions (*n* 1650).

Dependent variables	*n*	Mean	*SD*	**α**
Index F1 Women	1,498	2.51	0.88	0.91
Index F2 Women	1,479	2.35	0.88	0.83
Index F3 Women	1,485	1.78	0.96	0.80
Index F1 Men	1,503	2.60	0.86	0.90
Index F2 Men	1,477	2.05	0.76	0.72
Index F3 Men	1,484	1.56	0.88	0.73
Independent variables	*n*	Valid %		
**Age**				
16–20 (ref.)	358	21.7		
21–27	308	18.6		
28–50	365	22.1		
51–62	284	17.2		
63–74	338	20.4		
**Sex**				
Woman (ref.)	875	52.9		
Man	778	47.1		
**Education**				
Primary (ref.)	350	21.2		
Secondary	606	36.7		
Tertiary (<3 years)	241	14.6		
Tertiary (>3 years)	378	22.9		
**Country of origin**				
Swedish born (ref.)	1.456	88.1		
Foreign born	197	11.9		
**Resident municipality**
Medium sized	828	50.1		
Big city	544	32.9		
Rural area	281	17.0		
**Subjective class background**				
Working class (ref.)	775	46.9		
Self-employed and farmers	278	16.8		
White-collar service class	298	18.0		
White collar (higher/professionals)	194	11.7		
**My own occupation’s status/prestige**				
High (ref.)	391	23.7		
Medium	696	42.1		
Low	395	23.9		

**Table 5:** Determinants of social status perceptions.

	**Women’s social status**			**Men’s social status**		
	*F1 Index*	*F2 Index*	*F3 Index*	*F1 Index*	*F2 Index*	*F3 Index*
**Age** (ref. 16–20)						
21–27	–0.084*	–0.071^+^	–0.073^+^	–0.103*	–0.118**	–0.040
28–50	–0.119**	–0.147***	–0.060	–0.148***	–0.106*	0.016
51–62	–0.167***	–0.200***	–0.050	–0.189***	–0.149***	0.087*
63–74	–0.193***	–0.275***	0.003	–0.227***	–0.219***	0.121**
**Sex** (ref. women)						
Men	–0.060*	–0.142***	–0.112***	–0.039	–0.082**	–0.060*
**Education** (ref. primary school)						
Secondary school	0.053	0.010	0.024	0.082*	0.034	0.002
University (< 3 years)	0.093*	0.062^+^	0.085*	0.089*	0.075^+^	0.061
University (> 3 years)	0.179***	0.124**	0.061	0.208***	0.104^+^	0.046
**Country of origin** (ref. Swedish)						
Foreign born	0.056*	0.023	0.064*	0.072**	0.035	0.109***
**Place of residence** (ref. Big city)						
Medium-sized town	–0.030	–0.047^+^	0.014	–0.048^+^	–0.061*	0.032
Rural area	–0.055^+^	–0.059*	0.001	–0.102***	–0.064*	0.009
**Subjective class background** (ref. Working class)						
Self-employed/farmers	0.063*	0.040	0.040	0.048	0.056*	0.035*
White-collar service class	0.066*	0.009	–0.004	0.050	–0.001	0.014
White-collar professionals	0.083**	0.003	–0.017	0.090***	0.019	0.019
**Own occupation’s prestige** (ref. High status)						
Medium status	–0.004	0.019	–0.018	0.007	–0.016	–0.035
Low status	0.050^+^	0.089**	–0.019	0.069*	0.056^+^	–0.073*
**Constant**	2.58	2.66	1.86	2.68	2.17	1.46
**R**^2^ **adj**	0.061	0.096	0.017	0.078	0.049	0.038
**N**	1,498	1,479	1,485	1,503	1.477	1.484

^+^*p* < 0.1; *p < 0.05; **p < 0.01; ***p < 0.001 (Missing values replaced with means). OLS (standardized β coefficients).

According to Statistics Sweden, low response rates in survey studies have increased in recent years (Japec et al., 1997), risking data to be non-representative and lacking in credibility. The questions analyzed in this article are taken from a survey that partly replicates a previous one studying occupational prestige (Svensson and Ulfsdotter Eriksson, 2009). The occupational prestige scores from 2002 and 2018 shows an almost perfect correlation (ρ = 0,974; See Ulfsdotter Eriksson and Nordlander, 2024). The high correlation and the similarity in profiles of the raters from both studies, testify that the data have sufficient credibility for the exploratory purpose of this article. However, there is a slight under-representativeness in both surveys from low educated, low-paid, and foreign-born.

## 4 Findings

The findings are presented in two steps. First, we focus on the existence of underlying status dimensions and discuss their relative importance, as well as that of individual status qualifiers. Thereafter, we explore the variations in perceptions of the status dimensions found.

### 4.1 Social status dimensions and qualifiers

As Tables 3, 4 show, there are both similarities and differences in what is considered important for the social status of a woman and a man. The main similarity is the underlying dimensions (Factors 1–3), and their overall importance (grand means). The PCA solutions are almost identical in terms of the qualifiers that load strongly on the dimensions. The three dimensions have some correspondence with Bourdieu’s concepts of economic, cultural, and social capital, but it seems difficult to interpret them in that way. In addition, as this is not a deductive study, but rather an inductively and explorative one, we discuss more unconditionally how these dimensions may be understood and conceptualized.

Factor 1 is the most important one for both genders. There are surely economic aspects to this dimension, but it also includes qualifiers relating to cultural resources, such as occupation and education (*cf.* institutionalized cultural capital), residential area and housing type, and other social status possessions, such as cars, boats, or summer houses, which are all strong lifestyle indicators (*cf.* objective cultural capital). It is not that strange that property, occupation, and education load on factor 1, together with economic resources, since they are related to income and can be seen as prerequisites for or expressions of economic resources. Overall, the qualifiers in this dimension correspond to materialized achievements external to the individual, and it consists of indicative and categorical status cues (*cf.* Berger et al., 1998).

Factor 2 is the second most important one in terms of grand means while being closer in importance to Factor 1 for a woman’s than a man’s social status. The items loading on this factor indicate sociocultural resources, encompassing qualifiers such as manners (behavior and speech), looks and clothing style, having many friends and a large social network, marriage, and children, and – particularly for women’s status – ancestry and family name, indicate (embodied) sociocultural capital. This dimension concerns interactional and relational aspects of self-presentation and behaviors in social relations (Leary et al., 2014), and is seen as expressive in both tasks and categories (Berger et al., 1998).

Factor 3, illustrating more internally founded tastes and interests, is the least important for the status of both genders. The qualifiers loading on this dimension relate to both social and organizational capital. They encompass the extent to which one participates or shows an interest in civic associations and politics, nature and the environment, art, literature, classical music, or antiquities. While the last qualifier may seem to indicate cultural capital, the phrasing of the question as whether the respondent has “an interest in” may explain why it loads in this factor. In addition, these items may be defined as indicative and task-oriented status cues (Berger et al., 1998).

From the grand means, we see that the difference in importance between the three dimensions is greater for a man’s social status than for a woman’s social status. The status qualifier that is most dissimilar in terms of factor loadings between Tables 3 and 4 is ancestry/family name: whereas this qualifier loads mainly on Factor 2 for a woman’s social status, it loads moderately on both Factor 1 and 2 for a man’s social status.

Turning to the individual qualifiers, we find a high correlation between the two rankings of means in Tables 3 and 4 (ρ = 0.827***). Surprisingly, at the bottom of the list are traditional cultural practices such as interest in art, literature, classical music, and antiquities. These are thus not particularly strong social status cues in current Swedish society. All qualifiers included in Factor 3 are in fact placed at the bottom of the ranking of means (i.e., <2), indicating that the suggested Swedish version of organizational capital seems to confer less status than the other qualifiers. Being married and having children are also among the low-rated qualifiers.

At the top of the list, manners and speech are surprisingly highly regarded for the social status of both women and men. Occupation is the second most important qualifier generally – albeit superseded by looks and clothing style for a woman’s social status. The position of occupation is less surprising given that it has been considered “the backbone of social stratification” (Ganzeboom and Treiman, 1996, 202), and the one qualifier most important for the economic positioning of an individual.

It is noteworthy that the grand mean of all individual qualifiers is higher for a woman’s social status (2.29) than a man’s (2.18), and 10 of the 14 items have higher means for a woman. This finding is difficult to interpret, but it may indicate that it requires more for social status to be attributed to women. Women must qualify on more aspects than men to gain or signal status. Furthermore, four items show particularly marked differences, regarding both means and standardized mean comparisons (data not shown; see Section 3). Looks and clothing style, as well as marriage and children, are more important for a woman’s social status, whereas having money and financial assets and owning other kinds of property is more important for a man. There is an impact of gender-traditional views that economic resources, the external items, are more important for a man’s status, whereas outward appearance and having a family, the interactional ones, are more important for a woman’s status.

### 4.2 Determinants of social status perceptions

The next step is to analyze whether there are group-level differences in perceptions. As indicated by the adjusted R2 values and the standardized *β* coefficients (Table 5), the overall explanatory power of the models is rather low, but there are also significant effects to discuss. Age shows the strongest effects: that is, the younger the respondent, the more emphasis is on Factor 1, the *external* qualifiers, and Factor 2, the *interactional*, for the social status of either gender. Nevertheless, there are dissimilar effects on Factor 3 (the *internal*), as age has no effect on this dimension for a woman’s social status and a positive effect on a man’s social status. Material assets and materialized or personal achievements are thus more important for younger Swedes than for the elders, and cues related to a commitment to associations, the environment, and intellectual culture are more important for the older respondents, especially concerning a man’s social status.

The effects of sex make interpretation difficult because women rated importance on all dimensions higher for both genders, as compared to men. There may be a gender perception bias because this tendency is valid for all 14 items in individual regressions (data not shown). However, as the effects vary across the regressions, some conclusions may be drawn. First, regarding women’s social status, Factor 2, the dimension of interactional qualifiers, and to some extent Factor 3 (the internal), is more strongly emphasized by women than Factor 1, the dimension of external qualifiers. The effect of gender on the dimensions of a man’s social status is not that strong or varied, but we find a slight tendency for women to emphasize the interactional qualifiers for a man’s social status. Second, women emphasize all dimensions of a woman’s status more than a man’s status, compared with the importance men attribute to them. This might indicate that it requires more for a woman to gain social status.

The effects of education are particularly strong on Factor 1 for the status of both women and men. The higher the educational level of the respondent, the more emphasis is placed on the external qualifiers as an important status dimension. There is also a tendency for more educated respondents to put more weight on Factor 2, the interactional aspects.

Country of origin shows some effects. Foreign-born respondents emphasize Factor 3, the internal qualifiers, and to some extent also Factor 1, the externals, more than Swedish-born people for the social status of both sexes, whereas there are weaker and nonsignificant effects on Factor 2, the interactional qualifiers. Regressions on individual items (not shown) indicated that it was “interest in art literature, classical music, antiquities” that was rated as more important by foreign-born respondents, indicating that taste in the fine arts may not be as devalued in terms of status in other cultural contexts as in the Swedish one.

The effect of place of residence shows that respondents from large cities value the externals, Factor 1, more for a man’s social status than respondents from rural areas. There is a slight tendency for the same to apply to a woman’s social status, and the interactional (Factor 2) is more important for the social status of both women and men according to respondents from large cities. However, these effects are weak and have rather low levels of significance.

The subjective class has one significant effect: respondents who grew up in white-collar professional families tend to emphasize the importance of the external qualifiers (Factor 1) more than respondents from working-class backgrounds.

The final background variable, which is based on respondents’ subjective estimation of their own occupation’s prestige shows that individuals in low-status occupations seem to value the interactional qualifiers (Factor 2) for a woman’s social status, and the external qualifiers (Factor 1) for man’s social status more than respondents in high-status occupations, who instead put more emphasis than on the internal ones (Factor 3). These results indicate a slight tendency for gender-traditional views that economic resources are more important for men’s status whereas outward appearances and having a family are more important to strengthen women’s status among people in low-status occupations.

## 5 Discussion and conclusion

This study aimed to explore the importance of common social status qualifiers for the social status of a woman and a man. The findings add to the literature by increasing our understanding of how lifestyle factors affect individual social status in contemporary Sweden.

By analyzing the importance of 14 lifestyle-related qualifiers for social status, this study revealed three underlying dimensions, which have similarities but do not neatly fall into the Bourdieusian categories of economic, cultural, social, or organizational capital, as there are aspects of cultural resources in all three (*cf.* Bourdieu, 1984, 1986). Factor 1 gathers external qualifiers that strongly relate to economic and external resources, Factor 2 assembles qualifiers relating to social and interactional resources, and Factor 3 consists of qualifiers that signal cultural and organizational capital and internal resources.

Most important for social status for both genders are items of what one has in terms of external and material achievements, such as financial resources, education and occupation, residential area and housing type, and having other kinds of durable goods, such as summer houses, boats, or cars (*cf.* Carr and Vignoles, 2011; Leary et al., 2014). The second most important is related to interactional aspects, which include self-presentation in terms of manners, looks and clothing, friends, social networks, and being married with children. The least important of the three dimensions refers to internal aspects, covering interest and engagement in civic associations and politics, nature and the environment, or art, literature, classical music, and antiquities.

Further, there is a proximity between the three dimensions and the theory of status cue processes discussed by Berger et al. (1998). Factor 2 signifies interactional and self-presenting which relates to the concept of “categorial cues” representing “who these people are,” while Factor 3 relates to the things that individuals do, that is, “task cues” that indicate “what these people are doing and can do” (Fişek et al., 2005, 82). Factor 1, what one has, relates to both task and categorial cues, but points beyond them by also indicating what one has already achieved in terms of material success or failure. This surely has to do with the status cues process theory being developed for status signals in locally situated contexts, whereas this study explores status qualifiers at a more general social level.

The similarities between what is considered important for the social status of women and men are striking, both regarding the three underlying dimensions (Factor 1–3) and the ranking of the 14 status qualifiers. This may be related to Sweden being one of the most gender-equal countries in the world (WEF, 2019). Nevertheless, there are some gender-related differences of a very gender-stereotypical kind. Economically related resources and achievements, specifically having money and expensive property, are considered more important for a man’s social status. In contrast, the interactional status qualifiers, particularly looks, clothing style, and being married with children, are seen as more important for a woman’s status. Furthermore, the analysis indicated that a broader set of status qualifiers may be required for women than for men to achieve or signal high status because in general, most qualifiers are rated higher for women.

Regarding the determinants of social status perceptions, effects from the background variables exist but are not that strong or easy to interpret. Reduced to somewhat simplified formulas, the result shows that the status qualifiers comprising Factor 1 are emphasized by younger people, those with high levels of academic education, those who live in cities, people with upper-middle-class backgrounds, and foreign-born. Following Inglehart (2018), it is more surprising that the younger respondents who could be expected to express more postmaterialist values, instead seemed to lean toward more success-oriented achievements in terms of external and interactional status qualifiers. Factor 2 is particularly emphasized by women, especially in relation to their perceptions of a woman’s status. Factor 3 is emphasized more by older Swedish-born people and for men’s social status particularly by people in prestige occupations, irrespective of their class background or education.

These results have implications for the discussion of Sweden as a postmaterialist or even postmodern consumer society (Söderqvist-Tralau, 2009; Inglehart, 2018; Ulver, 2019). It may be seen as surprising that ownership and material achievements are so highly valued, and interests and civic orientations hold so much less value. On the other hand, in a society heavily oriented toward consumption, singularity, and socially oriented perfectionism, it may seem logical that external and material resources are of great importance (Curran and Hill, 2019; Ulver, 2019; Reckwitz, 2021). The postmaterialism thesis has not gone without criticism, and analyses show that this trend among youth has been reversed in many countries (*cf.* Abramson, 2011).

In short, our results indicate that Sweden is neither a fully postmodern nor postmaterialist society in the sense that social status is fully determined by consumer identities or that material assets play a minor role in social stratification. “Modern” status qualifiers, such as the possession of economic resources, a good education, and a prestigious occupation, still play a significant role. However, achievements and sociocultural capital related to identity, self-presentation, and having friends and networks also bring status. Even cultural resources related to postmaterialist engagement in civic associations, politics, or nature and the environment may bring social status, whereas the traditional highbrow cultural consumption practices of being interested in art, literature, classical music, or antiquities are of less importance. In addition, despite the striking similarities in how women and men are valued in terms of status, there are still remnants of rather gender-traditional views on social status, although this may be expected to be much stronger in other cultural and political contexts.

There are of course limitations to the generalizability of these results and conclusions. As the data are cross-sectional, arguments concerning temporal change, generational effects, and causality are impossible to make with certainty. Even more importantly, the effects of cultural context are only speculative because we have no country-comparative data. Therefore, the results must be developed and tested in future research. Another necessary line of comparison is to explore the status qualifiers, as different status groups may confer different statuses depending on factors such as clothing, neighborhood, housing, or car. That is, how specific status distinctions vary between groups and categories.

## Data availability statement

The datasets presented in this article are not readily available because we are restricted by the ethical regulation in Sweden and that we stated in our ethical application that (1) data sets will be kept in password protected computers and (2) that results reported in the scientific publications will only be described on an aggregated level. Requests to access the datasets should be directed to Ylva.Ulfsdotter.Eriksson@lnu.se.

## Ethics statement

The studies involving humans were approved by Regionala etikprövningsnämnden i Göteborg (Regional Ethics Review Board in Gothenburg). The studies were conducted in accordance with the local legislation and institutional requirements. Written informed consent for participation was not required from the participants or the participants’ legal guardians/next of kin because written information of the study was given in the survey questionnaire and consent given when sending in the questionnaire.

## Author contributions

YUE: Conceptualization, Data curation, Formal analysis, Funding acquisition, Investigation, Methodology, Project administration, Writing – original draft, Writing – review & editing. BL: Conceptualization, Data curation, Formal analysis, Methodology, Writing – original draft, Writing – review & editing.

## References

[ref1] Abramson P. R. (2011). Critiques and Counter-Critiques of the Postmaterialism Thesis: Thirty-four Years of Debate, UC Irvine: Center for the Study of Democracy. https://escholarship.org/uc/item/3f72v9q4

[ref2] Alexander C. N. Jr. (1972). Status perceptions. American Sociological Review 37, 767–773. doi: 10.2307/2093586

[ref5] Anderson C. John O. P. Keltner D. Kring A. M. (2001). Who attains social status? Effects of personality and physical attractiveness in social groups. Journal of Personality and Social Psychology 81, 116–132. doi: 10.1037/0022-3514.81.1.116 11474718

[ref6] Anderson C. Kraus M. W. Galinsky A. D. Keltner D. (2012). The local-ladder effect: Social status and subjective well-being. Psychological Science 23, 764–771. doi: 10.1177/0956797611434537 22653798

[ref3] Anderson C. Cowan J. (2014). “Personality and status attainment: A micropolitics perspective” in The psychology of social status, New York. eds. Cheng J. T. Tracy J. L. Anderson C.. New York: Springer, 99–117.

[ref4] Anderson C. Hildreth J. A. D. Howland L. (2015). Is the desire for status a fundamental human motive? A review of the empirical literature. Psychological Bulletin 141:574. doi: 10.1037/a0038781 25774679

[ref7] Atkinson W. (2018). The social space, the symbolic space and masculine domination: the gendered correspondence between class and lifestyles in the UK. European Societies 20, 478–502.

[ref8] Bauman Z. (2007). Consuming Life, Cambridge: Polity.

[ref9] Beck U. Beck-Gernsheim E. (2002). Individualization, London: Sage.

[ref10] Berger J. Webster M. Ridgeway C. Rosenholtz S. (1998). “Status cues, Expectations, and Behaviour” in Status, Power and Legitimacy. eds. Berger J. Zeldistch M.. New York: Routledge.

[ref11] Berggren H. Trägårdh L. (2022). The Swedish Theory of Love, Seattle: University of Washington Press.

[ref12] Berglund T. (2024). “Occupational Change and Inequality” in Scrutinising Polarisation. eds. Berglund T. Ulfsdotter Eriksson Y.. London: Routledge.

[ref13] Birenbaum A. (1984). Aging and housing: A note on how housing expresses social status. Journal of Housing for the Elderly 2, 33–40. doi: 10.1300/J081V02N01_04

[ref14] Blaker N. M. van Vugt M. (2014). “The status-size hypothesis: How cues of physical size and social status influence each other” in The psychology of social status, New York. eds. Cheng J. T. Tracy J. L. Anderson C.. New York: Springer, 119–137.

[ref15] Blau P. M. (1960). A theory of social integration. American Journal of Sociology 65, 545–556. doi: 10.1086/222785

[ref16] Bourdieu P. (1984). Distinction, London. Routledge & Kegan Paul Ltd.

[ref18] Bourdieu P. (1986). “The Forms of Capital” in Handbook of Theory and Research for the Sociology of Education. ed. Richardson J. G.. New York: Greenwood Press.

[ref19] Bourdieu P. (1991). Language and Symbolic Power, Cambridge: Polity.

[ref17] Bourdieu P. (2005). The Social Structures of the Economy, Cambridge: Polity.

[ref20] Bourdieu P. Wacquant L. J. D. (1992). An Invitations to Reflexive Sociology, Chicago: The University of Chicago Press.

[ref21] Bridge G. (2001). “Bourdieu, rational action and the time‐space strategy of gentrification”, Transactions of the Institute of British Geographers 26, 205–216. doi: 10.1111/1475-5661.00015

[ref23] Broady D. (1985). “Inledning” in Kultur och utbildning. ed. Broady D.. UHÄ: Stockholm.

[ref22] Broady D. (1998). Kapitalbegreppet som utbildningssociologiskt verktyg, Skeptron Occasional Papers, 15. Uppsala: ILU.

[ref24] Campbell C. (2004). “I Shop Therefore I Know that I Am: The Metaphysical Basis of Modern Consumerism,” in Elusive Consumption. eds. Ekström K. M. Brembeck H. (Oxford: Berg).

[ref25] Carr H. L. Vignoles V. L. (2011). Keeping up with the Joneses: Status projection as symbolic self‐completion. European Journal of Social Psychology 41, 518–527. doi: 10.1002/ejsp.812

[ref27] Chancel L. Piketty T. Saez E. Zucman G.. (2022). World Inequality Report 2022, World Inequality Lab wir2022.wid.world. Available at: https://wir2022.wid.world/wwwsite/uploads/2023/03/D_FINAL_WIL_RIM_RAPPORT_2303.pdf

[ref26] Chan T. W. (2011). Social Status and Cultural Consumption, Cambridge: Cambridge UP.

[ref28] Chan T. W. Goldthorpe J. H. (2007). Class and Status: The Conceptual Distinction and its Empirical Relevance. American Sociological Review 72, 512–532.

[ref29] Chan T. W. Birkelund G. E. Aas A. K. Wiborg Ø. (2011). Social Status in Norway. European Sociological Review 27, 451–468.

[ref30] Chiang Y. C. Chu M. Zhao Y. Li X. Li A. Lee C. Y.. (2021). Influence of subjective/objective status and possible pathways of young migrants life satisfaction and psychological distress in China. Frontiers in Psychology 12:612317. doi: 10.3389/fpsyg.2021.612317 34122214 PMC8187866

[ref31] Clark G. (2012). “What is the True Rate of Social Mobility in Sweden? A Surname Analysis”, 1700-2012, https://citeseerx.ist.psu.edu/viewdoc/download?doi=10.1.1.691.3645&rep=rep1&type=pdf

[ref32] Curran T. Hill A. P. (2019). Perfectionism is increasing over time: A meta-analysis of birth cohort differences from 1989 to 2016. Psychological Bulletin 145:410. doi: 10.1037/bul0000138 29283599

[ref33] De Botton A. (2008). Status Anxiety, New York: Vintage.

[ref34] De Graaf N. D. Flap H. D. (1988). With a little help from my friends: Social resources as an explanation of occupational status and income in West Germany, The Netherlands, and the United States. Social Forces 67, 452–472. doi: 10.2307/2579190

[ref35] Delhey J. Schneickert C. Hess S. Aplowski A. (2022). Who values status seeking? A cross-European comparison of social gradients and societal conditions. European Societies 24, 29–60. doi: 10.1080/14616696.2021.2005112

[ref36] Featherstone M. (1991). Consumer Culture & Postmodernism, London: Sage.

[ref37] Fişek M. H. Berger J. Norman R. Z. (2005). Status cues and the formation of expectations. Social Science Research 34, 80–102. doi: 10.1016/j.ssresearch.2003.10.004

[ref9003] Frevert T. K. Walker L. S. (2014). Physical attractiveness and social status. Sociology Compass 8, 313–323.

[ref38] Fujishiro K. Xu J. Gong F. (2010). What does “occupation” represent as an indicator of social status? Exploring occupational prestige and health. Social Science & Medicine 71, 2100–2107. doi: 10.1016/j.socscimed.2010.09.026 21041009

[ref39] Ganzeboom H. B. Treiman D. J. (1996). Internationally comparable measures of occupational status for the 1988 International Standard Classification of Occupations. Social Science Research 25, 201–239. doi: 10.1006/ssre.1996.0010

[ref40] Gidron N. Hall P. A. (2017). The politics of social status: Economic and cultural roots of the populist right. The British Journal of Sociology 68, 57–S84. doi: 10.1111/1468-4446.12319 29114868

[ref41] Goffman E. (1969). Stigma, Harmondsworth: Penguin.

[ref42] Haas A. Gregory S. W. (2005). The impact of physical attractiveness on women’s social status and interactional power. Sociological Forum 20, 449–471. doi: 10.1007/s11206-005-6597-2

[ref43] Häuberer J. (2014). Social Capital in Voluntary Associations. European Societies 16, 570–593. doi: 10.1080/14616696.2014.880497

[ref44] Inglehart R. F. (2018). Cultural Evolution. Cambridge: Cambridge U.P.

[ref9004] Japec L Ahtiainen A Hörngren J. (1997). Minska bortfallet. Stockholm: SCB.

[ref45] Karlsson L. (2017). Self-Placement in the Social Structure of Sweden: The Relationship between Class Identification and Subjective Social Placement. Critical Sociology 43, 1045–1061.

[ref46] Kim H. Y. Pettit N. C. (2015). Status is a four-letter word: Self versus other differences and concealment of status-striving. Social Psychological and Personality Science 6, 267–275. doi: 10.1177/1948550614555030

[ref47] Kim J. O. Mueller C. W. (1978). Factor analysis, London: Sage.

[ref48] Larsson B. Letell M. Thörn H. (eds) (2012). Transformations of the Swedish Welfare State, Basingstoke: Palgrave Macmillan.

[ref49] Leary M. R. Jongman-Sereno K. P. Diebels K. J. (2014). “The pursuit of status: A self-presentational perspective on the quest for social value” in The psychology of social status, New York. eds. Cheng J. T. Tracy J. L. Anderson C.. Springer, 159–178.

[ref50] Merton R. K. (1968). “Social Structure and Anomie,” in Social Theory and Social Structure. New York: Free Press.

[ref51] Milner M. (2019). “Status Distinctions and Boundaries” in Routledge Handbook of Cultural Sociology. eds. Grindstaff L. Minch-Chen M. L. Hall J. R.. London: Routledge.

[ref9005] Oesch D. Vigna N. (2022). A decline in the social status of the working class? Conflicting evidence for 8 Western countries, 1987–2017. Comparative Political Studies 55, 1130–1157.

[ref52] Podolny J. M. (2005). Status Signals, Princeton: Princeton UP.

[ref53] Reckwitz A. (2021). The End of Illusions, Cambridge: Polity

[ref54] Ridgeway C. L. (2019). Status, New York: Russell Sage Foundation.

[ref55] Sauder M. Lynn F. Podolny J. M. (2012). Status: Insights from Organizational Sociology. Annual Review of Sociology 38, 267–283. doi: 10.1146/annurev-soc-071811-145503

[ref57] Svensson L. G. Ulfsdotter Eriksson Y. (2009). Yrkesstatus, Göteborg: Göteborgs universitet.

[ref56] Söderqvist-Tralau M. (2009). Status, Stockholm: Norstedts.

[ref58] Therborn G. (2020). Sweden’s turn to economic inequality, 1982-2019. Structural Change and Economic Dynamics 52, 159–166. doi: 10.1016/j.strueco.2019.10.005

[ref59] Treiman D. J. (1977). Occupational Prestige in Comparative Perspective, New York: Wiley

[ref60] Turner B. S. (1988). Status, Minneapolis: University of Minnesota Press.

[ref61] Ulfsdotter Eriksson Y. Nordlander E. (2023). On the Discrepancy of Descriptive Facts and Normative Values in Perceptions of Occupational Prestige. Sociological Research Online 28, 716–735.

[ref62] Ulfsdotter Eriksson Y. Nordlander E. (2024). “Occupational prestige and gendered polarization” in Scrutinising Polarisation. eds. Berglund T. Ulfsdotter Eriksson Y.. London: Routledge.

[ref9006] Ulver S. (2012). Status. Malmö: Liber.

[ref63] Ulver S. (2019). “Market Wonderland: An Essay about a Statist Individualist Consumer Culture” in Nordic Consumer Culture. eds. Askegaard S. Östberg J.. Basingstoke: Palgrave Macmillan.

[ref64] Weber M. (1970). From Max Weber, London: Routledge & Keegan Paul.

[ref65] Weber M. (1978). Economy and Society (vol 1), Berkeley: UCLA Press.

[ref66] WEF (2019). Global Gender Gap Report 2020, Geneva: World Economic Forum.

[ref67] Wegener B. (1992). Concepts and measurement of prestige. Annual Review of Sociology 18, 253–280. doi: 10.1146/annurev.so.18.080192.001345

[ref68] Whitbeck L. B. Hoyt D. R. (1994). Social prestige and assortative mating: a comparison of students from 1956 and 1988. Journal of Social and Personal Relationships 2, 137–145. doi: 10.1177/0265407594111008

[ref69] Wilkinson R. G. Pickett K. (2009). The spirit level, London: Allen Lane.

